
JOURNAL INFORMATION
==============================
NLM Title Abbreviation: ScientificWorldJournal
Journal ID: ScientificWorldJournal
NLM Journal ID: 101131163
Journal ID: TSWJ

The Scientific World Journal

ISSN: 2356-6140
EISSN: 1537-744X
Publisher: Wiley

ARTICLE INFORMATION
==============================
PMCID: PMC5956448
PMID: 15349527
DOI: 10.1100/tsw.2004.46
Article ID: 769610
Article version: 1
Subjects: Research Article

Gastrocystoplasty

Carr Michael C.
Mitchell Michael E.
University of Washington School of Medicine, Children's Hospital and Regional Medical Center, USA
Academic Editor: Anthony Atala

Publication date: 2004
Electronic publication date: 2004 Jun 7
Volume: 4
First page: 48
Last page: 55
Copyright: Copyright © 2004 Michael C. Carr and Michael E. Mitchell.
Copyright year: 2004
License: This is an open access article distributed under the Creative Commons Attribution License, which permits unrestricted use, distribution, and reproduction in any medium, provided the original work is properly cited.
License URL: https://creativecommons.org/licenses/by/3.0/

==============================
Article
*TheScientificWorldJOURNAL* (2004) 4 (S1), 48–55
ISSN 1537-744X; DOI 10.1100/tsw.2004.46

### Gastrocystoplasty

Michael C. Carr, M.D., Ph.D. and Michael E. Mitchell, M.D.
*University of Washington School of Medicine, Children's Hospital and Regional Medical Center*

**E-mails**: mcarr@email.chop.edu

*Previously published in the Digital Urology Journal*

**DOMAIN**: urology

Augmentation cystoplasty has been described using ileum, cecum or sigmoid colon,[1-3] as isolated flaps or in various shapes, combinations and quantities. This experience led to the creation of large and compliant urinary reservoirs, such as the Mainz,[4] Koch,[5] Indiana[6] or ileal neobladder.[7] Enterocystoplasties and the creation of urinary reservoirs from bowel are not always possible or contributed to metabolic derangements. Innovative work demonstrated that the stomach could be used to augment the bladder successfully.[8,9] This paved the way for the application of the gastrocystoplasty to the pediatric population.[10]

The use of stomach in the bladder facilitates net excretion of chloride ions, which in turn supports a main buffer system of the urine (ammonium chloride). This permits secretion of acid without the use of titratable acids that potentially leads to the depletion of the buffer systems. Ammonia resorption does not occur either, as has been observed when large and small bowel are used in the urinary tract. In a partial renal failure model, gastrocystoplasty actually protected the dogs with chronic renal failure from the acidosis of chronic ammonium chloride loss.[11] Theoretically, a patient with chronic acidosis and renal insufficiency could be protected, in part, by gastrocystoplasty, whereas large and small bowel in the same capacity would only serve to increase the acidotic state. This has proven to be the case clinically. The presence of acid in the urine may also contribute to reduced infection. In the rat model metaplastic changes have been noted, but the potential for malignant degeneration is still uncertain.

The use of the stomach has proven to be invaluable in reconstructive procedures for patients with major complex problems. We have applied it to patients in whom no other type of bowel was available for urinary reconstruction. This included those with previous radiation and those with absence of large bowel or reduced small bowel (the cloacal exstrophy group). Stomach has also been selected for patients with metabolic acidosis, as this condition would be exacerbated by the addition of large or small bowel because of chloride or ammonia resorption. The aciduria could prove beneficial for patients with chronic and recurrent infection.

Despite the successes with gastric augmentation and bladder substitution, the ideal augmentation would not introduce either bowel or gastric mucosa into the urinary tract. Metabolic complications, urolithiasis, mucus production, spontaneous perforation, and even malignant degeneration would be eliminated. A urothelial-lined bladder with gastrointestinal tract muscle augmentation of the detrusor muscle was attempted as early as 1955.[12] Other demucosalized intestinal segments have also been used in several other studies.[13,14] Another surgical attempt to preserve transitional epithelium is detrusorrhaphy or autoaugmentation.[15] The detrusor, however, is potentially replaced with fibrous tissue or whatever adheres to urothelium, which may result in inadequate bladder volume or poor compliance. An autoaugmentation gastrocystoplasty or demucosalized gastric flap procedure (DAWG) uses a full thickness urothelial graft in conjunction with a raw inner surface of incorporated stomach muscle/submucosa.[16]

### OPERATIVE TECHNIQUE

Patient selection for gastrocystoplasty is no different than for any bladder reconstruction patient. Initially, patient selection was limited to those patients that had a deficiency of bowel or patients with metabolic acidosis. The procedure is well suited, however, to patients that use small catheters or who, in general, would tolerate mucus and highly viscous urine poorly, and for patients with chronic urinary infection problems.

Rigorous bowel preparation is generally not required. Most patients will be placed on a liquid diet 48 hours before surgery in addition to receiving a bottle of Magnesium Citrate 24 hours prior to surgery. Mechanical and antibiotic bowel preparations have been used in patients anticipating major reconstruction or who have had multiple previous abdominal procedures. Recently, because of restrictions on hospital days, patients have been admitted the day of surgery. When possible, all patients will have preoperative video urodynamics, a renal ultrasound, and either an IVP or renal scan (MAG-3 lasix renogram or DMSA scan). Every effort is made to sterilize the urine prior to the procedure. In children we have not felt it necessary to obtain an upper gastrointestinal series. However, in the adult this may be prudent.

A long midline incision is made from the symphysis pubis to the xyphoid process. A self-retaining ring retractor is very useful in this procedure which demands proper exposure deep in the pelvis and high in the epigastria. In the case of gastrocystoplasty the bladder is initially explored through only the lower portion of the incision and opened in sagittal plane in the midline from the bladder neck (anteriorly) to the trigone (posteriorly). The upper portion of the incision and peritoneum usually need to be opened only after the bladder preparation is complete. Ureteral reimplantation is performed at this time, if necessary. It is also a good time to develop a plane around the bladder neck should this be necessary (as for implantation of an artificial sphincter, for a bladder neck suspension or bladder neck wrap[17]). Pediatric feeding tubes are left in the ureters to facilitate identification and prevent injury during the augmentation. The bladder is packed off with a moist sponge and the upper abdomen explored.

The stomach is brought well into the surgical field. It is helpful to use large Babcock clamps to do this maneuver (be sure that the Babcock clamps do not compromise or injure the gastroepiploic arteries). The gastroepiploic artery (GEA) is then carefully evaluated. Common observations at this point will be that the gastroepiploic artery along the left inferior margin of the greater curvature is either very thin or will apparently dive into the stomach wall. Thus, it is usually preferable to base the wedge gastric flap on the right GEA. The greater omentum is then incised parallel to the GEA several centimeters inferior to this vessel. The electrocautery and bipolar electrode can be used to do this very effectively. Larger vessels, however, may need to be ligated. Although some of the omental vessels are quite significant and it may seem preferable to base the wedge flap on these vessels, we have not done so to date as it would necessitate bringing the blood supply of the flap anterior to the abdominal contents. The triangular gastric wedge is next selected with the apex of the wedge close to, but not including the lesser curvature of the stomach. The length along the greater curvature is usually about 9-15cm in length, but this depends upon the patient's age, size and anticipated final bladder volume. The position of the wedge will be dependent on the anticipated blood supply. If the more constant right GEA pedicle is used, the wedge is usually taken closer to the cardia of the stomach. The rule of thumb is that the largest possible wedge should be taken with the longest blood supply without altering gastric physiology. Usually about a third to half of the stomach is removed. The short arteries from the gastroepiploic to the stomach flap are kept intact. The others along the anticipated pedicle are carefully ligated in place and divided (Figure 1A). Great care must be taken during this portion of the procedure to make sure that the GEA is protected and not injured.

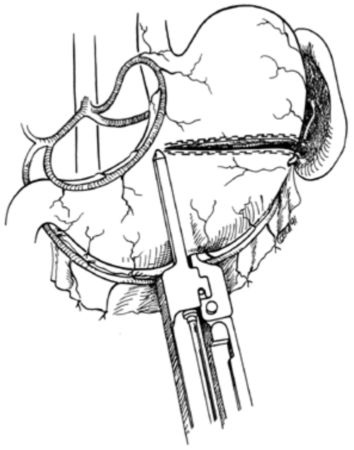

**FIGURE 1A**

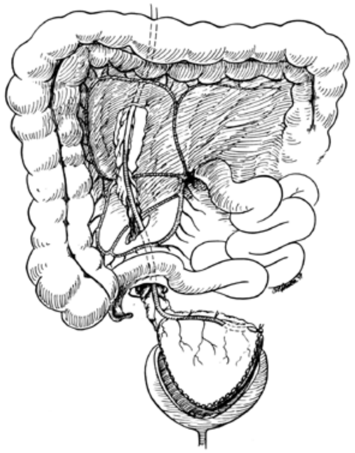

**FIGURE 1B**

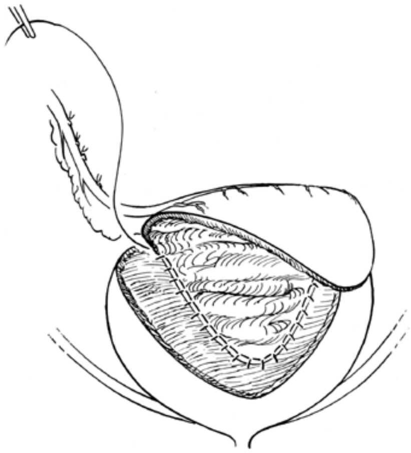

**FIGURE 1C**

It is sometimes helpful to gently apply papaverine solution on the artery along its entire length to prevent spasm. The GEA at the distal end of the wedge is then divided. The 90mm GIA stapler is used to resect the wedge. The staples in place prevent blood loss from the flap and stomach during the repair of the stomach. The wedge flap is wrapped in a moist sponge and placed in the pelvis with a moist sponge over the pedicle to protect it during the gastric repair. The repair of the stomach resection is made in two layers. An outer layer of interrupted 3-0 silk sutures in the muscularis and serosa and an inner layer of through and through running 3-0 Monocryl suture is used. The posterior interrupted silk sutures are placed before the gastric lumen is opened. The staples may be left in the posterior suture lines. A nasogastric tube is then positioned across the suture line and this will be left in place until bowel motility is appreciated (this usually occurs postoperative day four or five). The wedge-shaped gastric flap is then brought with its blood supply through the mesentery of the transverse colon and through the root of the small bowel mesentery (Figure 1B). It, therefore, courses in a retroperitoneal position with its blood supply on the right side. Make sure that there is no twisting of this pedicle in the process of placing the flap on the bladder. The wedge-shaped flap should reach well down into the pelvis without tension on the blood supply. If further length of the pedicle is required, this is secured by dissecting proximally at the gastroduodenal angle.

The wedge flap is then opened to form a parallelogram-shaped flap. All the staples are removed. The posterior apex is then sutured into the area of the posterior bladder wall close to the trigone (Figure 1C). Running locking through and through 3-0 Moncryl sutures are then used to sew the back wall in place. A second layer of 3-0 Vicryl sutures through the muscularis and serosa then ensure a watertight anastomosis. A Malecot catheter is usually place before the anterior segment of the augmentation is sutured in place. The size of this catheter depends on the age of the patient. It is usually, however, preferable to have greater than a 14 French tube. This tube is usually brought out through native bladder if possible, but it may exit through the flap if fixed to the abdominal wall like a gastrostomy tube. The ureteral catheters are left in place during the posterior anastomosis and then may be removed if reimplantation or tapering has not been performed. The anterior portion of the augmentation is then completed and the bladder is tested to make sure that the suture lines are watertight. Any leaks must be repaired at this point. We tend not to drain the gastrocystoplasty patients. The omentum, which is still intact, is placed over the anterior portion of the bowel after the windows in the retroperitoneum have been closed with running chromic suture. With the omentum tucked over the bowel and behind the augmented bladder the bladder resides in a position that is close to its original position. A careful check should be made that there is no tension on the pedicle of the flap.

### TOTAL BLADDER REPLACEMENT WITH THE WEDGE FLAP

Total bladder replacement with stomach is possible using the wedge flap. Following removal of the staples, the apex of the wedge is simply sutured to the urethra (Figure 2). This may be feasible in the patient with an intact urethra (bladder replacement). The urethra may be sutured to the apex of the flap which can be tubularized to provide a nice bladder neck. Several options are available for patients requiring construction of a catheterizable reservoir. A distal ureter may be used as a catheterizable channel. The ureter can be tunneled in between mucosa and muscularis of the reservoir wall to provide continence. This is generally possible only in the cloacal exstrophy population. The appendix, as well, has been used in a similar manner. The construction of a bladder tube, using a strip of the gastric flap, has been used successfully (Figure 3A). The tube is nippled into the reservoir to provide continence. This has resulted in a channel which is catheterizable and dry (Figure 3B). Our preference, however, would be to use a tunnel technique for dryness with the construction of a gastric reservoir. In bladder replacement it is usually most convenient to open the wedge completely and suture the posterior tip (which usually is the anterior apex of the wedge) onto the urethra. Subsequently, with the gastric segment open, ureteral reimplantation is easily performed in much the same way that a tunneled reimplant would be performed in the bladder. A plane between the mucosa and muscularis of the gastric flap is usually easily defined with sharp dissection. The anterior portion apex is then sewn to the anterior portion of the urethra and the lateral aspects of the wedge are then closed using a two layer closure of running 3-0 Monocryl and Vicryl. A suprapubic tube (Malecot catheter) may be brought through the gastric tissue, but if such is done, it would be advisable to suture the wall of the gastric reservoir to the anterior abdominal wall as would be the case with a gastrostomy. This prevents intraperitoneal leakage when the tube is subsequently removed. A catheterizable stoma should be constructed flush with adjacent tissue and in such a manner that catheterization can be performed without difficulty. A small V-flap of skin and spatulation of the catheterizable channel works well. If catheterization is difficult in the operating room, it will be impossible out of the operating room.

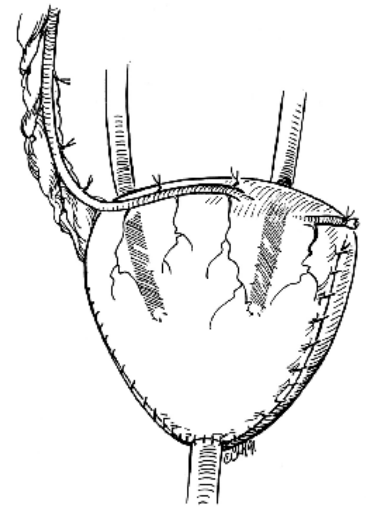

**FIGURE 2**

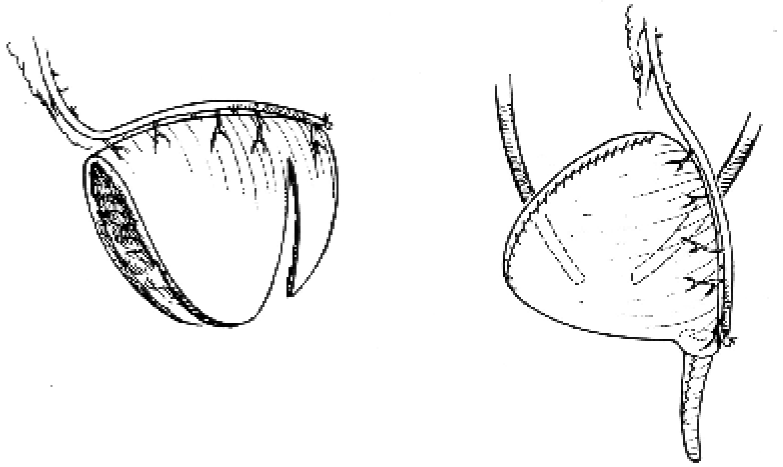

**FIGURES 3A and 3B**

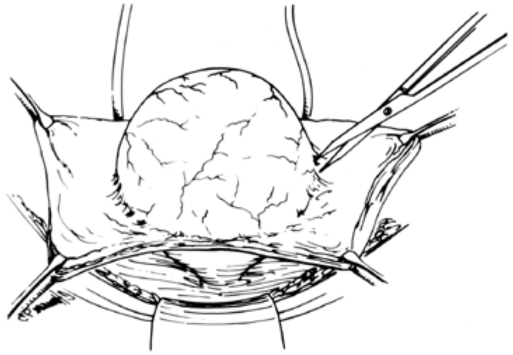

**FIGURE 4A**

### DEMUCOSALIZED GASTRIC FLAP PROCEDURE

Augmentation of a bladder after detrusorrhaphy with demucosalized gastric flap is similar to gastrocystoplasty. The bladder is approached initially. It is helpful to have a Foley catheter in the bladder connected to a reservoir of irrigating saline that can be raised or lowered to fill or empty the bladder as desired. The bladder is filled to a pressure of approximately 20cm of water and the detrusor incised in the midline sagittal plane. Great care is taken to preserve the transitional epithelium in finding the plane between the transitional epithelium and muscularis. It is easiest to use blunt and sharp dissection with the bladder full to do this (Figure 4A). If no holes are made in the epithelium, this usually goes very easily. Once a hole is made and the bladder is decompressed it becomes very difficult to dissect the plane between the transitional epithelium and the bladder muscularis. We usually remove muscularis from two-thirds to three-quarters of the anterior and lateral wall of the bladder. The bladder is emptied by lowering the reservoir, followed by gentle packing with moist sponges. An alternative technique is to use a large balloon catheter (Helmstein balloon) to facilitate this dissection. This can be left in place following the surgery to promote healing of the epithelium to the seromuscular flap.

A gastric flap is obtained. Before it is brought into the pelvis, however, the mucosa is removed. This is done very easily by removing the staples and placing four quadrant sutures for traction so the flap is opened as a parallelogram. A vascular loop is gently placed around the pedicle with enough traction so that the blood supply to the pedicle is temporarily interrupted. It takes approximately ten to fifteen minutes to sharply dissect the mucosa from the muscularis. Potts tenotomy scissors are used for this purpose. The proper plane, which is easily found, tends to have a somewhat foamy appearance (Figure 4B). Once the mucosa is removed the pedicle vascular loop is loosened and bleeders are electrocauterized using the bipolar cautery. Most of the bleeding will be in the midline where the perforators follow the gastroepiploic pedicle. The demucosalized flap is brought into the pelvis as with any augmentation. The demucosalized flap is then laid directly on top of the outside of the bladder epithelium raw surface to raw surface. Either a Penrose drain or a suction drain is then placed between the transitional epithelium and flap and brought out through the abdominal wall. This will be removed in approximately 48 hours. The demucosalized gastric flap is sutured in place using a running, locking 3-0 Vicryl suture as a single layer closure (Figure 4C). In an effort to keep the contact between the transitional epithelium and the demucosalized gastric flap the bladder is kept partially filled at approximately 20cm water pressure using a vented drainage system. This system is maintained for approximately five to seven days. Alternatively, a large ballooned catheter (Helmstein) may be used for this purpose. Subsequently, the patient is placed on either intermittent catheterization or intermittent clamping of a suprapubic tube. If a suprapubic tube is used in the patient, it is brought through the intact bladder wall rather than through the healing transitional epithelium and demucosalized flap.

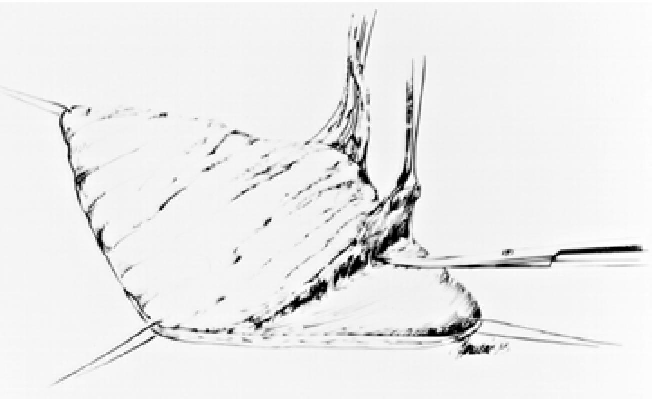

**FIGURE 4B**

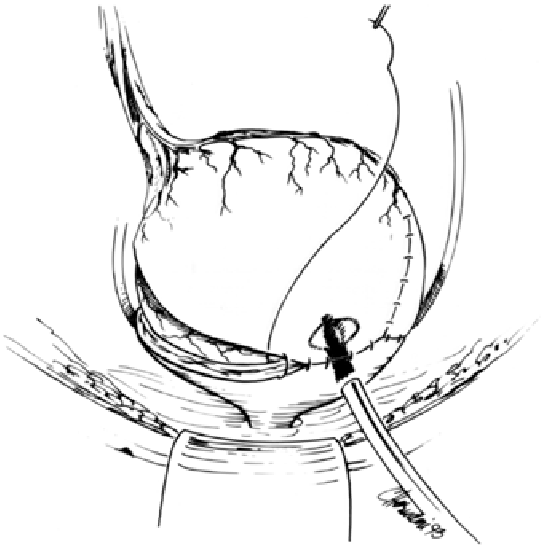

**FIGURE 4C**

### POSTOPERATIVE CARE

The management of the gastrocystoplasty patient is similar to that of any patient with intestinal augmentation. Mucus is generally not a problem, but irrigation of the bladder should be routinely performed initially. All patients are maintained on intravenous antibiotics and then converted to chronic suppression for at least two months after surgery. $H_2$-blockade, usually consisting of Ranitidine (1mg/kg q8h), is continued for one to two months postoperatively. If the patient then develops dysuria or perineal pain, the Ranitidine is resumed until the symptoms resolve or the hematuria improves. Removal of the suprapubic tube is dependent on the ability of the patient to empty the bladder. A trial period of at least one week of continuous clamping is usually wise while the patient begins to void or intermittent catheterization is reinstated. Finally, patients with anuria, or dilute urine, may rarely require irrigation with phosphate buffer if low pH and irritation is observed. Infection is less common with the gastrocystoplasty patient, but asymptomatic infection is usually not treated unless the organism is felt to be significant for potential stone formation or pyelonephritis. Serum electrolytes should be followed periodically.

### EARLY AND LATE COMPLICATIONS

Our initial experience in 80 patients with stomach augmentation or replacement in bladder has been very encouraging. With an average follow-up of six years all but three patients have shown either stable or improved renal function. All but 7 patients are dry and urodynamics have demonstrated a marked improvement in compliance. Gastrin levels have not been elevated in our group and no patient has experienced duodenal or gastric ulceration. Early complications have included gastric bleed in one patient which did not require transfusion, a perivesical extravasation in one patient and an obstruction of a ureter reimplanted into the stomach in one patient. Delayed complications have included bowel obstruction in seven patients, spontaneous bladder perforation in three patients and hematuria and/or dysuria in 28 patients. The so-called hematuria/dysuria syndrome is unique to the use of stomach in the urinary tract.[18] The symptoms are usually intermittent, self-limiting and improve with time. The spectrum of severity is seen ranging from intermittent dysuria and tea colored urine to the rare occurrence of ulcer formation and bladder perforation. The majority of patients are controlled with intermittent use of $H_2$ blockers, sodium bicarbonate bladder irrigation or oral bicarbonate therapy. A few children have required short-term use of oral Omeprazole, (an antisecretory compound that suppresses gastric acid secretion). The symptoms are more pronounced in adult patients and those children with sensate urethras in whom voiding is attempted or if there is wetness. The etiology is believed to be due to hydrochloric acid production by the gastric segment and reduced buffering effect by urine. However, symptoms have been seen even with urine acidity in the normal range. An alternative explanation proposed is that the irritation and potential ulceration is due to Helicobacter pylori, the same pathogen that leads to gastric ulcers. There is a risk for developing a peptic ulcer in the gastric portion of a defunctionalized gastrocystoplasty. Anuric states (i.e., renal failure or urinary diversion) should be treated with basic bladder irrigations such as phosphate buffered saline or bicarbonate to prevent this.

Patients with gastrocystoplasties who lose large amounts of hydrochloric acid, sodium and potassium in the urine may become severely dehydrated and alkalotic. This usually occurs only when patients have significant salt losses from the bowel or kidney such as with severe diarrhea or emesis, salt-losing nephropathy or rarely hypergastrinemia. The ensuing hyponatremic hypochloremic alkalosis requires intravenous saline volume replacement and is usually easily corrected. It may be that excessive salt loss related to increased serum gastrin relates to reduced acid production in the stomach secondary to gastric resection. This can sometimes be reversed with carbonic acid (soda pop) with meals. The majority (77%) of the patients on clean intermittent catheterization have had at least one positive urine culture.

Our follow-up of patients who have undergone the demucosalized gastric flap procedure is limited to ten patients. All patients have tolerated the procedure well. Postoperative urodynamics will be necessary in order to demonstrate improvement in bladder capacity and compliance, but our early urodynamic data is very encouraging. For those patients who do not have a problem with recurrent urinary tract infections or have underlying renal insufficiency, this procedure may be an excellent alternative which avoids some of the complications of the traditional gastrocystoplasty.

Our experience with use of stomach for urinary reconstruction now extends to more than 80 patients treated at this institution. The diagnoses include neurogenic bladder, bladder exstrophy, exstrophy of the cloaca and urethral valves and ectopic ureter. We have performed augmentation and bladder replacement in adults and children although most of our experience is with the younger patients. Stomach can be used to totally replace the bladder, however, older male patients can experience urethral irritation. The advantages are little mucus, reduced infection and the ability to easily tunnel ureters or a conduit as a catheterizable stoma which make this a very appealing technique. We feel that in many clinical situations it is clearly advantageous over other tissues, particularly large and small bowel.

### REFERENCES

1. Goodwin, W.E., Turner, R.D. and Winter, C.C.: Results of ileocystoplasty. J Urol, 80:461, 1958.
2. Mitchell, M.E. and Piser, J.A.: Intestinoplasty and total bladder replacement in children and young adults: follow-up in 129 cases. J Urol, 138:579, 1987.
3. Hendren, W.H. and Hendren, R.B.: Bladder augmentation experience with 129 children and young adults. J Urol, 144:445, 1990.
4. Thuroff, J.W., Alken, P., Engelmann, J., Riedmiller, H., Jacobi, G.H. and Hohenfellner, R.: The Maiz pouch (mixed augmentation with ileum and cecum) for bladder augmentation and continent diversion. J Urol, 136:17, 1986.
5. Skinner, D.G. Lieskovsky, G., Boyd, S.: Continent urinary diversion. J Urol, 141:1323, 1989.
6. Rowland, R.G., Mitchell, M.E., Bihrle, R., Kahnoski, R.H. and Piser, J.E.: Indiana continent urinary reservoir. J Urol, 137:1136, 1987.
7. Hautmann, R.E., Egghart, G, Frohneberg, D and Miller, K.: The ileal neobladder. J Urol, 139:39, 1988.
8. Sinaiko, E.S.: Artificial bladder in man from segment of stomach. Surg Forum, 8:685, 1957.
9. Leong, C.H.: Use of the stomach for bladder replacement and urinary diversion. Ann R Coll Surg Engl, 60:283, 1978.
10. Adams, M.C., Mitchell, M.E., Rink, R.C.: Gastrocystoplasty: an alternative solution to the problem of urologic reconstruction in the severely compromised patient. J Urol, 140:1152, 1988.
11. Kennedy, H.A., Adams, M.C., Mitchell, M.D., et al: Chronic renal failure and bladder augmentation: stomach versus sigmoid colon in the canine model. J Urol, 140:1138, 1988.
12. Shoemaker, W.C. and Marucci, H.D.: The experimental use of seromuscular grafts in bladder reconstruction: preliminary report. J Urol, 73:314, 1955.
13. Oesch, I.: Neourothelium in bladder augmentation: an experimental rat model. Eur Urol, 14:328, 1988.
14. Gonzalez, R, Busor, H., Reid, C. and Reinberg, Y.: Seromuscular colocystoplasty lined with urothelium: experience with 16 patients. Urology, 45:124, 1995.
15. Cartwright, P.C. and Snow, B.W.: Bladder autoaugmentation: early clinical experience. J Urol, 142:505, 1989.
16. Nguyen, D.H., Mitchell, M.D., Horowitz, M., Bagli, D.J. and Carr, M.C.: Demucosalized augmentation gastrocystoplasty with bladder autoaugmentation in pediatric patients. J Urol, 156:206, 1996.
17. Walker, R.D., Flack, C.E., Hawkins-Lee, B., Lim, D.J., Parramore, H., Hackett, R.L.: Rectus fascial wrap: early results of a modification of the rectus fascial sling. J Urol, 154:771, 1995.
18. Nguyen, D.H., Bain, M.A., Salmonson, K.L., Ganesan, G.S., Burns, M.W., Mitchell, M.E.: The syndrome of dysuria and hematuria in pediatric urinary reconstruction with stomach. J Urol, 150:707, 1993.

**This article should be referenced as follows:**

Carr, M.C. and Mitchell, M.E. (2004) Gastrocystoplasty. *TheScientificWorldJOURNAL* **4 (S1)**, 48–55.

**Handling Editor:**

Anthony Atala, Principle Editor for *Urology* — a domain of *TheScientificWorldJOURNAL*.
